# Estimating the Burden of False Positives and Implementation Costs From Adding Multiple Single Cancer Tests or a Single Multi‐Cancer Test to Standard‐Of‐Care Screening

**DOI:** 10.1002/cam4.70776

**Published:** 2025-03-17

**Authors:** Sarina Madhavan, Allan Hackshaw, Earl Hubbell, Ellen T. Chang, Anuraag Kansal, Christina A. Clarke

**Affiliations:** ^1^ Massachusetts General Hospital Boston Massachusetts USA; ^2^ GRAIL, Inc. Menlo Park California USA; ^3^ University College of London London UK

**Keywords:** blood‐based testing, cancer prevention, cancer screening

## Abstract

**Background:**

Blood‐based tests present a promising strategy to enhance cancer screening through two distinct approaches. In the traditional paradigm of “one test for one cancer”, single‐cancer early detection (SCED) tests a feature high true positive rate (TPR) for individual cancers, but high false‐positive rate (FPR). Whereas multi‐cancer early detection (MCED) tests simultaneously target multiple cancers with one low FPR, offering a new “one test for multiple cancers” approach. However, comparing these two approaches is inherently non‐intuitive. We developed a framework for evaluating and comparing the efficiency and downstream costs of these two blood‐based screening approaches at the general population level.

**Methods:**

We developed two hypothetical screening systems to evaluate the performance efficiency of each blood‐based screening approach. The “SCED‐10” system featured 10 hypothetical SCED tests, each targeting one cancer type; the “MCED‐10” system included a single hypothetical MCED test targeting the same 10 cancer types. We estimated the number of cancers detected, cumulative false positives, and associated costs of obligated testing for positive results for each system over 1 year when added to existing USPSTF‐recommended cancer screening for 100,000 US adults aged 50–79.

**Results:**

Compared with MCED‐10, SCED‐10 detected 1.4× more cancers (412 vs. 298), but had 188× more diagnostic investigations in cancer‐free people (93,289 vs. 497), lower efficiency (positive predictive value: 0.44% vs. 38%; number needed to screen: 2062 vs. 334), 3.4× the cost ($329 M vs. $98 M), and 150× higher cumulative burden of false positives per annual round of screening (18 vs. 0.12).

**Conclusions:**

A screening system for average‐risk individuals using multiple SCED tests has a higher rate of false positives and associated costs compared with a single MCED test. A set of SCED tests with the same sensitivity as standard‐of‐care screening detects only modestly more cancers than an MCED test limited to the same set of cancers.

## 1 Introduction

**FIGURE 1:**
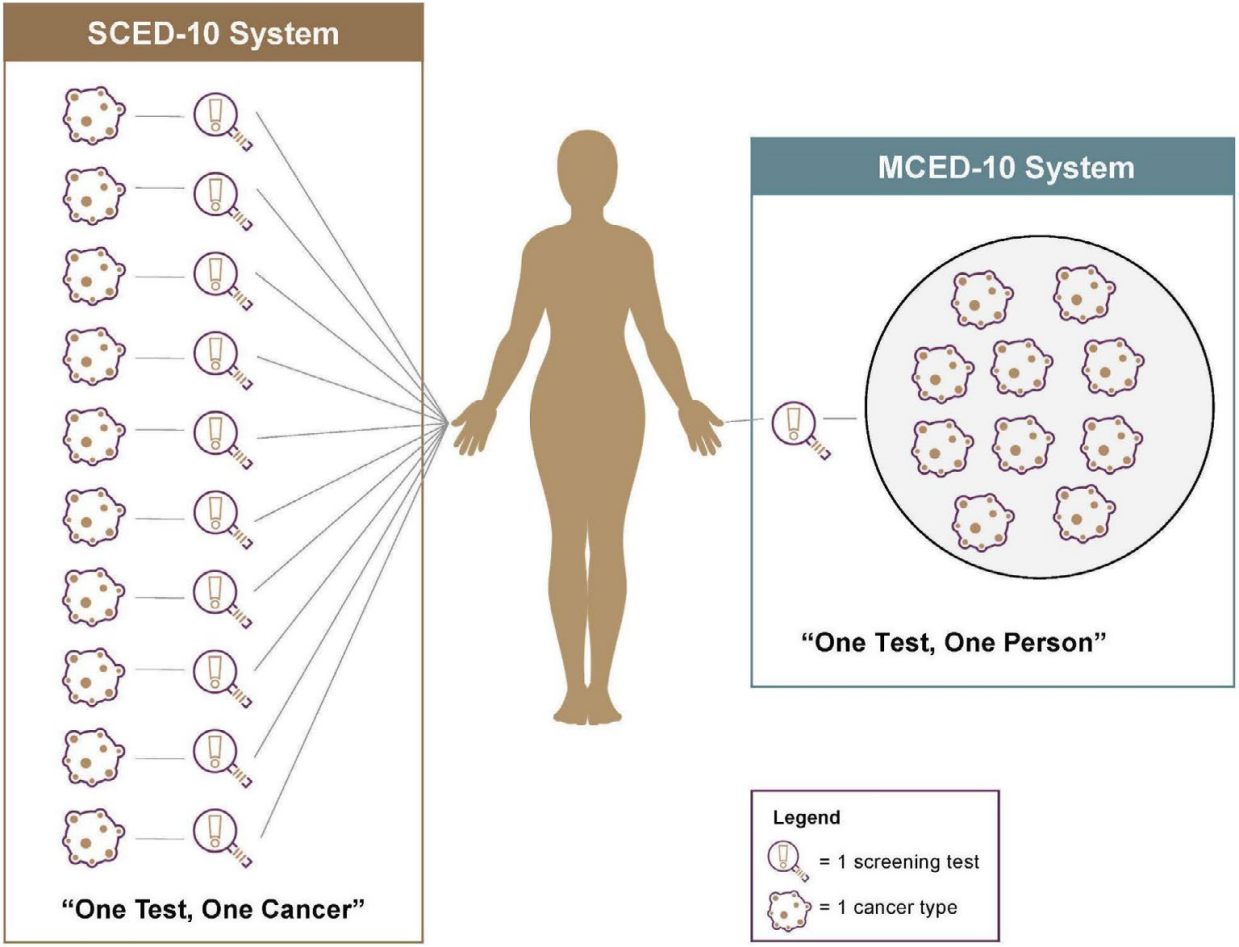
Schematic illustrating the concept of single‐cancer early detection (SCED) based on one test per cancer, and multi‐cancer early detection (MCED) based on one test per individual person.

The rapid emergence of both single‐cancer and multi‐cancer blood‐based screening tests compels a reassessment of the established evaluation framework for cancer screening technologies. These blood‐based tests hold the promise to identify many of the ~85% of cancers not covered or detected by guideline‐recommended screening [1, 2]. Single‐cancer early detection (SCED) and multi‐cancer early detection (MCED) tests present two distinct approaches for improving cancer outcomes through early diagnosis (Figure 1).

SCED tests have similar performance characteristics to existing cancer screening modalities, with single‐cancer true positive rates (TPR) for an individual cancer (typically > 75%) and higher false positive rates (FPR, typically 5%–15%) [1]. SCED tests reflect the traditional paradigm of “one test for one cancer” and can also target individuals with higher pre‐test risk (e.g., genetic predisposition) for specific cancers. For example, Guardant Health recently published a high‐sensitivity DNA‐based SCED blood test for colorectal cancer with performance characteristics similar to colonoscopies [3].

In contrast, MCED tests simultaneously target multiple cancers with a single, fixed, low FPR (< 1% and a corresponding specificity of > 99%) at the cost of a relatively lower TPR (ranging from 30% to 50% for all covered cancer types) [4, 5, 6, 7]. MCED tests prioritize population and individual‐level impact, particularly for average‐risk populations. Representative examples with ongoing clinical trials include Galleri (GRAIL Inc., Menlo Park, California) and CancerSEEK (Exact Sciences Corporation, Madison, Wisconsin) [5, 6].

The similarity between SCED tests and preexisting screening technologies makes them easily comparable. However, the strategy of MCED testing is not commensurate with the traditional approaches to screening of “one test for one cancer” and requires a new method for characterizing and comparing to the old regime. This paradigm shift in cancer screening warrants moving away from narrow evaluations of each individual test's technical performance and broadening to a high‐level view of the strategic challenges associated with delivering screening tests to patients. These challenges include, but are not limited to, the cumulative burden of false positives and overdiagnosis, total healthcare costs and resource allocation, and feasibility of integration into existing healthcare systems and workflows. While the use of both SCED and MCED tests is not mutually exclusive, these implementation challenges and costs likely impact their optimal use [8, 9].

Here, we developed a framework for examining and comparing the practical considerations associated with SCED and MCED cancer screening (Figure 1). Many have already modeled how MCED tests can achieve clinically meaningful endpoints like reduced mortality [10, 11, 12], and clinical trials for many such tests are ongoing [13]. Few studies have examined factors related to implementation in real terms, such as testing efficiency, affordability, and the impact of false positive results. To our knowledge, none have compared the implementation of an MCED screening approach to that of an SCED screening approach. We used established measures of screening system performance [14] to examine general population‐level health impacts of hypothetical SCED and MCED cancer‐screening approaches when added to existing United States Preventive Services Task Force (USPSTF) guideline‐recommended screening. The objective of this analysis was to compare the efficiency of screening systems in these two hypothetical scenarios. We did not conduct a cost‐effectiveness analysis because we do not yet have estimates of the effect of MCED tests on cancer mortality; this would merit a separate study with multiple sensitivity analyses using several cost items. Our aim here is to show the general principle of multiple screening tests on screening performance.

Analyzing each of these approaches to blood‐based cancer screening tests as systems enables researchers, clinicians, and healthcare administrators to gain a more complete understanding of how these tests could transform cancer screening and early detection paradigms. It also addresses complex challenges such as integration into existing healthcare systems, implementation feasibility across diverse clinical settings, as well as healthcare economics and resource allocation. By creating a framework for comparing the different approaches in blood‐based screening, we can better assess their potential to revolutionize cancer detection.

## 2 Methods

### 2.1 Reference Scenario Design Using Current Guideline‐Recommended Screening

We calculated the real‐world impact of USPSTF A and B guideline‐recommended screening, accounting for screening adherence and the individual sensitivities of each test [15]. This scenario was calculated to provide a reference scenario for system‐level (or “program‐level”) metrics associated with the widely accepted paradigm of single cancer screening. To calculate the impact of guideline‐recommended screening, we used adherence data from the US Behavioral Risk Factor Surveillance System (BRFSS) [16, 17], lung cancer screening eligibility data from the US National Health Interview Survey [18], and a Chicago screening cohort [19] (conservatively using a relatively high estimate of the proportion of lung cancers eligible for screening).

### 2.2 Hypothetical Scenario Design for SCED and MCED Systems

We compared two hypothetical scenarios implementing DNA‐based blood test screening as applied to the general US population in addition to the current standard of care, represented by the reference scenario described above.

The first hypothetical scenario represented the cumulative impact of adding 10 individual SCED tests (labeled “SCED‐10”), where each SCED test can identify one of the 10 cancer types responsible for the highest absolute number of cancer deaths in the United States (i.e., lung and bronchus, breast, colon and rectum, pancreas, liver and intrahepatic bile duct, esophagus, uterine corpus, bladder, lymphoma, and brain and nervous system) [2]. For this scenario, we excluded prostate cancer and leukemia given the complexities of liquid cancer and prostate cancer screening paradigms. Within this scenario, we assumed each person receives only the relevant tests (i.e., 10 per female, 7 per male).

The second hypothetical scenario represented the impact of a single MCED test per person targeting the same aforementioned 10 cancer types (labeled “MCED‐10”).

The impacts of the hypothetical SCED and MCED systems were expressed as incremental to USPSTF guideline‐recommended screening. In other words, in our analysis, any potential overlap between USPSTF‐recommended screening and SCED or MCED screening was attributed to USPSTF‐recommended screening alone; thus, all estimated impacts of SCED or MCED screening were above and beyond (i.e., incremental to) those of USPSTF‐recommended screening, with no overlap.

### 2.3 Data Inputs

Using Surveillance, Epidemiology, and End Results (SEER) cancer incidence data from 17 geographic regions from 2006 to 2015, when cancers were uniformly staged according to the 6th edition of the American Joint Committee on Cancer classification [20], we simulated these hypothetical screening systems being administered to a US population of 100,000 adults (50,000 men and 50,000 women) aged 50–79 years, consistent with the age groups eligible for USPSTF‐recommended screening. Current USPSTF guidelines recommend screening for female breast (mammography, typically at ages 50–74 years), cervical (cytology and high‐risk human papillomavirus, at ages 21–65 years), and colorectal (different tests are available, at 45–79 years) cancers based on age criteria alone. Lung cancer screening using low‐dose computed tomography is recommended for adults aged 50–80 years with a specified smoking history [15]. For USPSTF‐recommended screening, we restricted the eligible population to overlap the modeled age interval of 50–79 years in each case. These inputs are summarized in Table S1.

### 2.4 Assumptions

Characteristics of each system are summarized in Table 1; key screening test performance assumptions used in the calculations are summarized in Table S2. The SCED‐10 system consisted of 10 hypothetical SCED blood tests, each with a TPR (87%) and FPR (11%) comparable to the performance characteristics of screening mammography and of SCED tests in development, which have a wide range of FPRs [21, 22]. For example, Guardant Health reported a TPR of 83% and an FPR of 10% for their SCED test for colorectal cancer [3], and Cologuard (Exact Sciences Corporation, Madison, WI), a commercially available noninvasive stool‐based test, has an FPR of 13% [23]. We used the FPR for mammography (11%) because it is approximately midway in the FPR range for approved cancer screening tests (5%–15%) [1] and because mammography is a well‐established and widely accepted screening test. The MCED‐10 system used a single hypothetical MCED blood test based on Klein et al. 2021 [5], which describes a commercially available MCED test that can detect more than 50 cancer types. For simplicity of comparison, we limited the TPR of the hypothetical MCED‐10 system to the same 10 cancer types as SCED‐10, while preserving the FPR of the commercially available MCED test, though a narrower MCED might have a lower FPR. This study reported an overall FPR of 0.4% and provides TPRs for each cancer type and associated cancer stage (Table S3) [24]. The reported test performance of the MCED test included smoothing to ensure non‐decreasing TPR by stage and was standardized to a SEER population of adults aged 50–79 years [5].

**TABLE 1:** System parameters for modeling SCED‐10 and MCED‐10 screening SYSTEMS. Additional inputs and assumptions used in this analysis are presented in the Supporting Information, including Tables S1–S4.

	Reference scenario: USPSTF	Scenario 2: incremental SCED‐10 screen for 10 cancer types	Scenario 32: incremental MCED‐10 screen for 10 cancer types
Aggregate TPR	Test‐dependent	87%^a^ (no stage‐specific TPR)	63%^b^ (with stage‐specific TPR)
Aggregate FPR	Test‐dependent	62.3%^a^	0.50%
Performance	100%	100%	100%
Adherence	Test‐dependent	100%	100%
Cost^c^	Test‐dependent	$100 per test $1000 per individual female $700 per individual male $850 per average individual	$1000 per test $1000 per individual

Abbreviations: FPR, false positive rate; MCED, multi‐cancer early detection; SCED, single‐cancer early detection; SEER, Surveillance, Epidemiology, and End Results; TPR, true positive rate.

^a^ TPR and FPR of the SCED system are calculated by aggregating the performance specifications of 10 individual hypothetical blood tests, each with the same performance characteristics as mammography.

^b^ Aggregate TPR in a population where targeted cancer incidence was 507 per 100,000 individuals, based on SEER adults aged 50–79 years, and FPR was 0.5%.

^c^ All individuals within the MCED systems receive the same test, whereas men and women receive different sets of tests in the SCED system.

**FIGURE 2:**
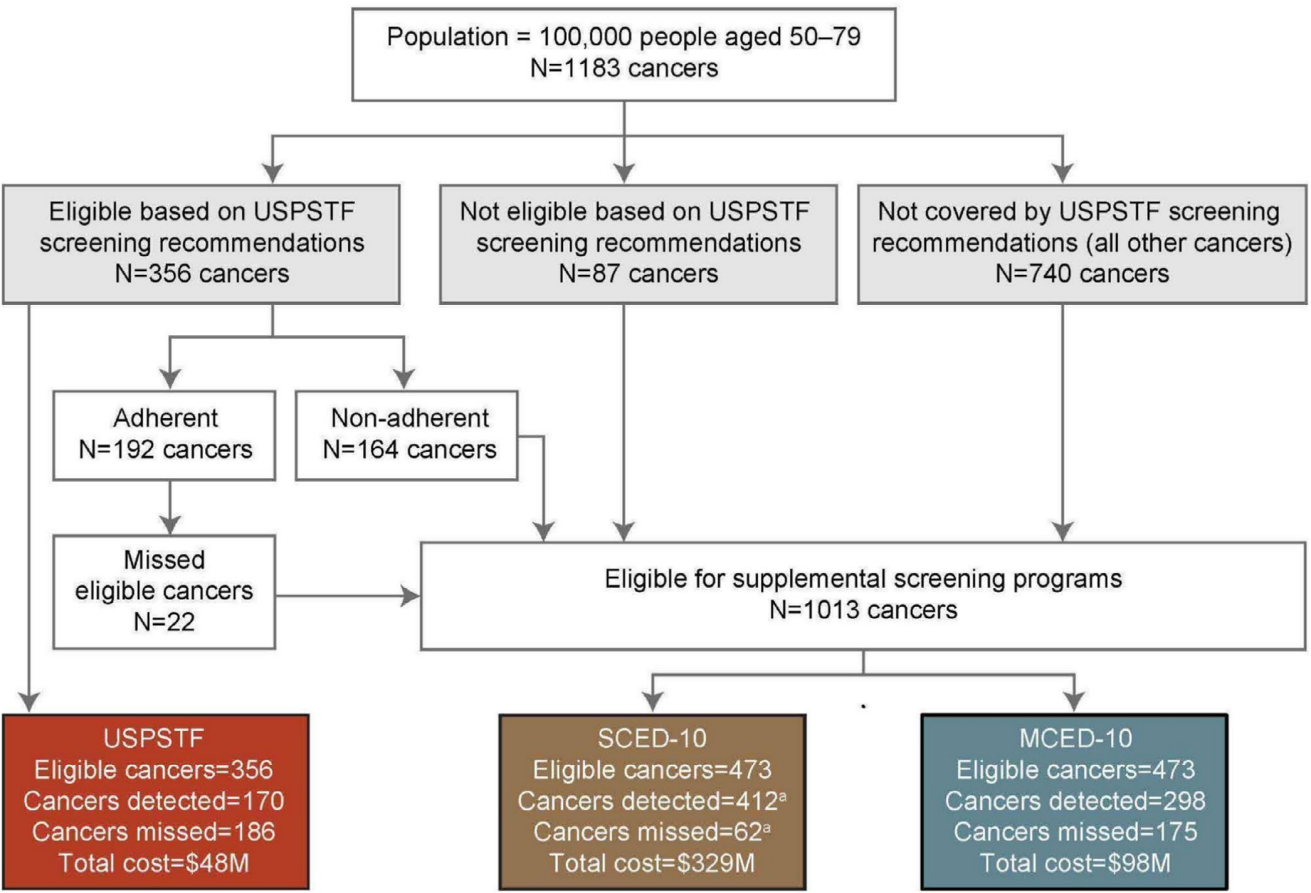
Overview of screening system performance modeling process. Screening pathway and outcomes for cancers detected by current recommended standard‐of‐care screening by USPSTF A & B recommendations, SCED‐10, and MCED‐10 for an average use population of 100,000 adults aged 50–79 years. The initial number of cancers (*N* = 1183) is based on SEER cancer incidence data for adults aged 50–79 years in 17 US geographic regions in 2006–2015. Colored boxes show the total number of cancers that can be detected by each screening system (“Eligible Cancers,” including 4 cancer types for USPSTF, and 10 cancer types for SCED‐10 and MCED‐10), are detected or not detected by each screening system based on test performance characteristics (“Cancers Detected” and “Cancers Missed”), and the total costs of each screening system (“Total Cost”) in the hypothetical population of 100,000 adults, accounting for USPSTF guideline‐based screening eligibility, adherence, and performance, with additional SCED or MCED screening as supplemental to USPSTF‐recommended screening. ^a^Counts do not sum to 473 due to rounding. Abbreviations: M, million; MCED, multi‐cancer early detection; SCED, single‐cancer early detection; SEER, Surveillance, Epidemiology, and End Results; USPSTF, United States Preventive Services Task Force.

Finally, the reported false positives of the MCED‐10 are distributed across cancer types in order to approximate the investigation costs assigned to individuals for false positives. Figure 2 outlines the modeling analysis and overview of outcomes. We assumed that all individuals received the same MCED‐10 test, whereas men and women receive different sets of tests under the SCED‐10 scenario and under current screening guidelines, as some screening tests are sex‐specific.

Both hypothetical scenarios (SCED‐10 and MCED‐10) were limited to the same 10 specific cancer types to provide a fair comparison. However, MCED technology may be designed to detect cancer signatures shared across multiple cancer types [5] and, as such, may not necessarily be configured to detect only a specific set of cancers and not others. Therefore, we calculated the incremental impact of a third scenario using the performance of a commercially available MCED test for more than 50 cancer types, Galleri® (“MCED‐G”) [5]. These calculations are described in the Supporting Information.

For the USPSTF scenario, we did not impute an average TPR given the differences in how TPR is calculated across each test and the differences in the definition of a cancer event across tests (see Supplemental Methods for additional clarification). Performance for currently recommended screening tests (for female breast, colorectal, lung, and cervical cancers) was based on the reported TPR and FPR of each test [22, 23, 24, 25]. We selected studies with higher estimates of screening uptake and assumed an annual frequency of mammography based on American Cancer Society guidelines (as opposed to biennial, based on USPSTF guidelines) in order to increase the number of cancers detected by current screening, and thus reduce the number available to be detected by supplemental screening. For the supplemental SCED‐10 and MCED‐10 screening scenarios, we assumed 100% adherence (in line with standard screening model assumptions) to assess the incremental benefit to screening program participants [26, 27] and one round of testing to reflect screening over a one‐year period.

Diagnostic investigations consisted mainly of imaging and biopsies and were taken to be cancer specific. In our calculations, we treated diagnostic investigations as driven by the patient's cancer type (summarized in Table S4) and did not model any effect for predictions of cancer origin, which are only provided by some MCED systems. Therefore, as described in the Supporting Information, we used generic costs, which are typically dominated by nonspecific cancer diagnostic investigations, to enable the broadest application of this model [28]. Test costs were assumed to be $100 per SCED‐10 test, conservatively assuming the low end of 2D mammography, which ranges from $107–$471, and of blood‐based colorectal cancer screening, which ranges from $100–$200 [29, 30]. Test costs for MCED‐10 test were assumed to be $1000, for the sake of simplicity and given the currently published prices of the MCED test referenced in Klein et al. [5] is $949. Cost calculations apply a 1.5 multiplier to the costs of cancer investigations following positive MCED test results, which is a strategy proposed by cost‐effectiveness analyses of USPSTF‐recommended cancer screening to help better capture competing risks [31, 32]. One paper modeling the cost‐effectiveness of colorectal cancer screening explored the role of cost 1×, 1.25×, and 1.5× multipliers and suggested that the 1.5× multiplier could better reflect potential future increases in medical expenses and uncertainties, making the screening strategies more robust to economic shifts [32]. Thus, we favored the higher end of these cost multipliers to better capture real‐world cost variability, providing a more conservative and comprehensive evaluation and to ensure that our analysis reflects screening under even unfavorable conditions that account for rising medical costs and future uncertainties [32].

The test performance for each screening scenario was applied to the simulated population of 100,000 adults to produce several standard cancer screening efficiency outcomes. More detailed descriptions of the calculations are found in the Supporting Information.

All outputs from our calculations represent system‐level (or “program‐level”) results distinct from individual‐level (or “test‐level”) results [33]. For simplicity, natural history effects, such as durations (dwell times) by cancer type and stage, were not incorporated into testing, and we assumed that the screening interval was well chosen for each test (see Supporting Information). However, system or program TPR incorporates many elements, including test TPR, population coverage/eligibility, and screening uptake/adherence. To enable comparisons across screening systems, the denominator for all screening system performance metrics is the entire population of adults in a specified age range during a specified time period. Thus, for instance, the FPR of the SCED‐10 scenario screening system (an output of our modeling) is distinct from the FPRs of the individual SCED tests (test‐level inputs into the modeling) that comprise the system.

### 2.5 Outputs

Screening efficiency and workload were quantified using the number of cancers detected, the number of diagnostic investigations in people without cancer, positive predictive value (PPV), aggregate negative predictive value (NPV, computed as the product of the individual cancer type NPVs, with the number of cancers targeted by each screening system as the denominator), all‐cancer NPV (computed in the same manner as aggregate NPV, but with the number of cancers of all types as the denominator), the number needed to screen (NNS) to find one incremental cancer, true‐positive rate (TPR) of the screening system, the FPR of the screening system, and costs of screening (i.e., testing and diagnostic investigations) under each screening scenario, averaged per year, in a typical screening‐age population ages 50 to 79 years. An incremental cancer is one found by an SCED or MCED test in addition to those found by USPSTF‐recommended screening tests.

We also calculated the average number of cumulative false‐positive test results experienced by an individual over their lifetime of screening under each scenario, assuming one test per year with each incremental test. For example, someone aged 50 years at the time of their first SCED/MCED test could be expected to undergo 30 of each individual test up to the age of 79 years.

### 2.6 Role of the Funding Source

This study was funded by GRAIL Inc.

### 2.7 Computation

Computational modeling was performed in R (code/data available at github link https://github.com/grailbio‐publications/Madhavan_Screening_Systems).

## 3 Results

**FIGURE 3:**
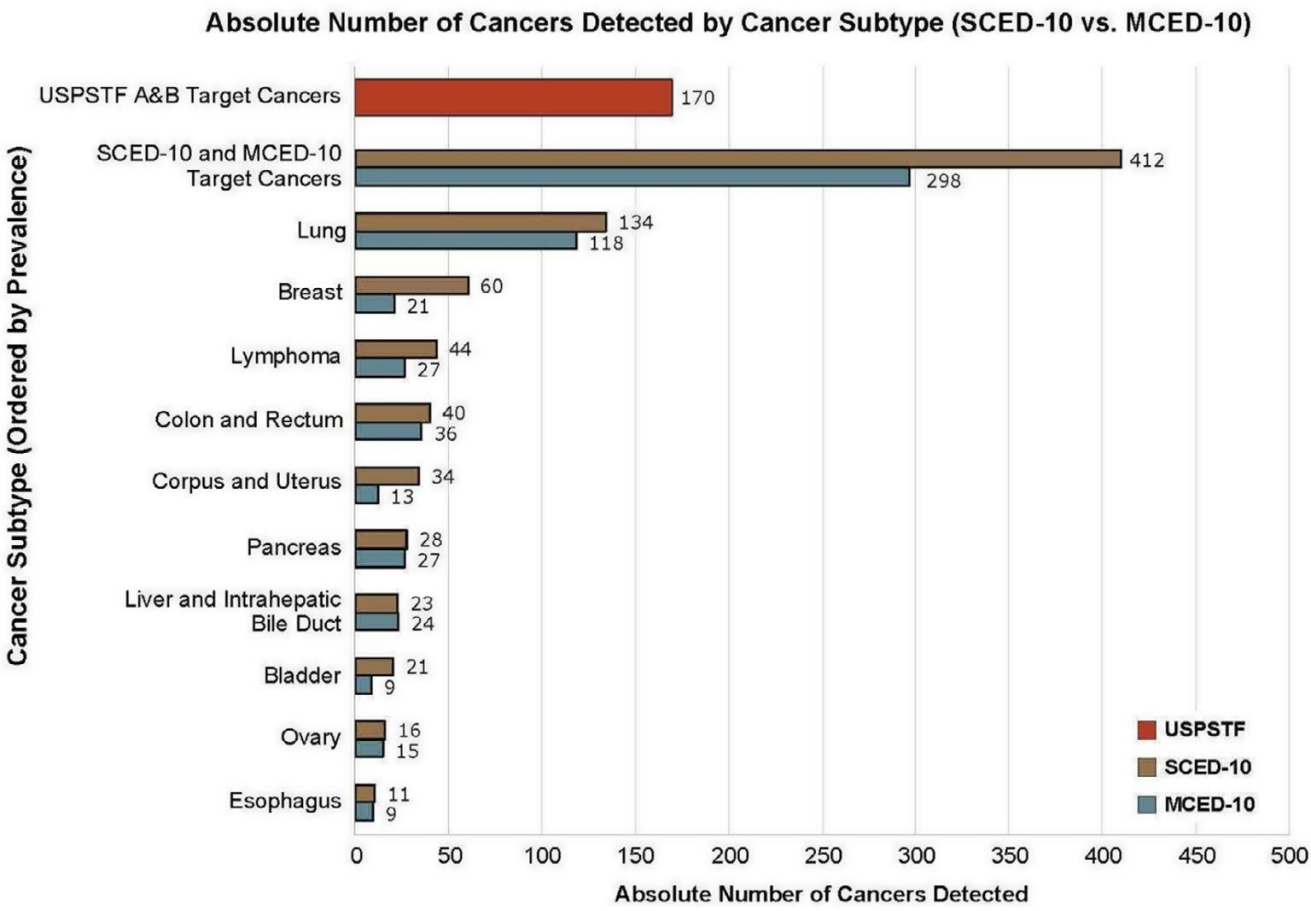
Incremental cancers by type detected using SCED‐10 versus MCED‐10 systems, with total cancers detected by USPSTF‐recommended real‐world reference screening system in 100,000 average‐risk adults aged 50–79 Years. Abbreviations: MCED, multi‐cancer early detection; SCED, single‐cancer early detection; USPSTF, United States Preventive Services Task Force.

In 100,000 US adults aged 50–79 years, 356 are expected to have cancer of the breast, colon, lung, or cervix each year in our modeling. Current USPSTF‐recommended screening tests have the potential to detect 356 cancers (female breast, colon, lung, and cervical) per 100,000 adults in a year, but are estimated to detect 170 due to screening eligibility, adherence, and test TPR (Table 2). The SCED‐10 screening system detected 412 incremental cancers (i.e., in addition to the number found by USPSTF‐recommended screening), while the MCED‐10 system detected 298 cancers. The SCED‐10 system found 38% more cancers than MCED‐10 (412 vs. 298), but with 188 times more additional diagnostic investigations in cancer‐free people, i.e., false‐positive test results (93,289 vs. 497; Table 2). Cancers detected by the SCED‐10 and MCED‐10 systems per cancer type are illustrated in Figure 3.

**TABLE 2:** Cancers detected, diagnostic investigations in people without cancer, and ratio per year for USPSTF, SCED‐10, and MCED‐10 screening systems among 100,000 average‐risk^a^ adults aged 50–79 years.

	Reference scenario: USPSTF	Scenario 1: incremental SCED‐10 screen for 10 cancer types	Scenario 2: incremental MCED‐10 screen for 10 cancer types
Total # of cancers eligible	356	473	473
Total # of tests performed	62,524	848,558	99,830
Cancers detected (% of total cancers eligible)	170 (48%)	412 (87%)	298 (63%)
Diagnostic investigations in people without cancer (% of Total Tests)	7496 (12%)	93,289 (11%)	497 (0.5%)
Ratio of cancers detected to diagnostic investigations in people without cancer	0.02	0.004	0.60
People without cancer receiving diagnostic investigations (% of Population)	7304 (7.3%)	62,167 (62.2%)	497 (0.5%)

Abbreviations: MCED, multi‐cancer early detection; SCED, single‐cancer early detection; USPSTF, United States Preventive Services Task Force.

^a^ In this context, “average‐risk” includes all adults aged 50–79 years and those for whom USPSTF recommends low‐dose computed tomography screening for lung cancer, i.e., adults in this age group who have a 20 pack‐year smoking history and currently smoke or have quit within the past 15 years.

Table 3 shows various measures of efficiency across the various screening systems; calculations by screening test type and screen‐eligible population are provided in the Supporting Information. The incremental PPV was more than 80‐fold lower with SCED‐10 than with MCED‐10 (0.44% vs. 37.53%; Table 3). The NNS was more than 6 times lower with MCED‐10 (334) than with SCED‐10 (2062; Table 3). Aggregate NPV, which is interpreted as the predictive value of a negative test result for the cancer types that are specifically targeted by a given screening system (ignoring the occurrence of other, untargeted cancer types), reflects different denominators based on the number of cancer types addressed by each system. All‐cancer NPV, which is interpreted as the predictive value of a negative test result for any cancer (accepting that all people are at risk for any cancer type in relevant organs), provides a fair comparison across screening systems based on the same denominator of all cancer types combined.

**TABLE 3:** Efficiency metrics of USPSTF, SCED‐10, and MCED‐10 screening systems in 100,000 average‐risk adults aged 50–79 years.

	Reference scenario: USPSTF	Scenario 1: incremental SCED‐10 screen for 10 cancer types	Scenario 2: incremental MCED‐10 screen for 10 cancer types
Positive predictive value	2.21%	0.44%	37.53%
Aggregate negative predictive value (for targeted cancer types)	99.8%	99.9%	99.8%
All‐cancer negative predictive value (for all cancer types)	99.0%	99.4%	99.3%
Number needed to screen	369	2062	334

Abbreviations: MCED, multi‐cancer early detection; SCED, single‐cancer early detection; USPSTF, United States Preventive Services Task Force.

**FIGURE 4:**
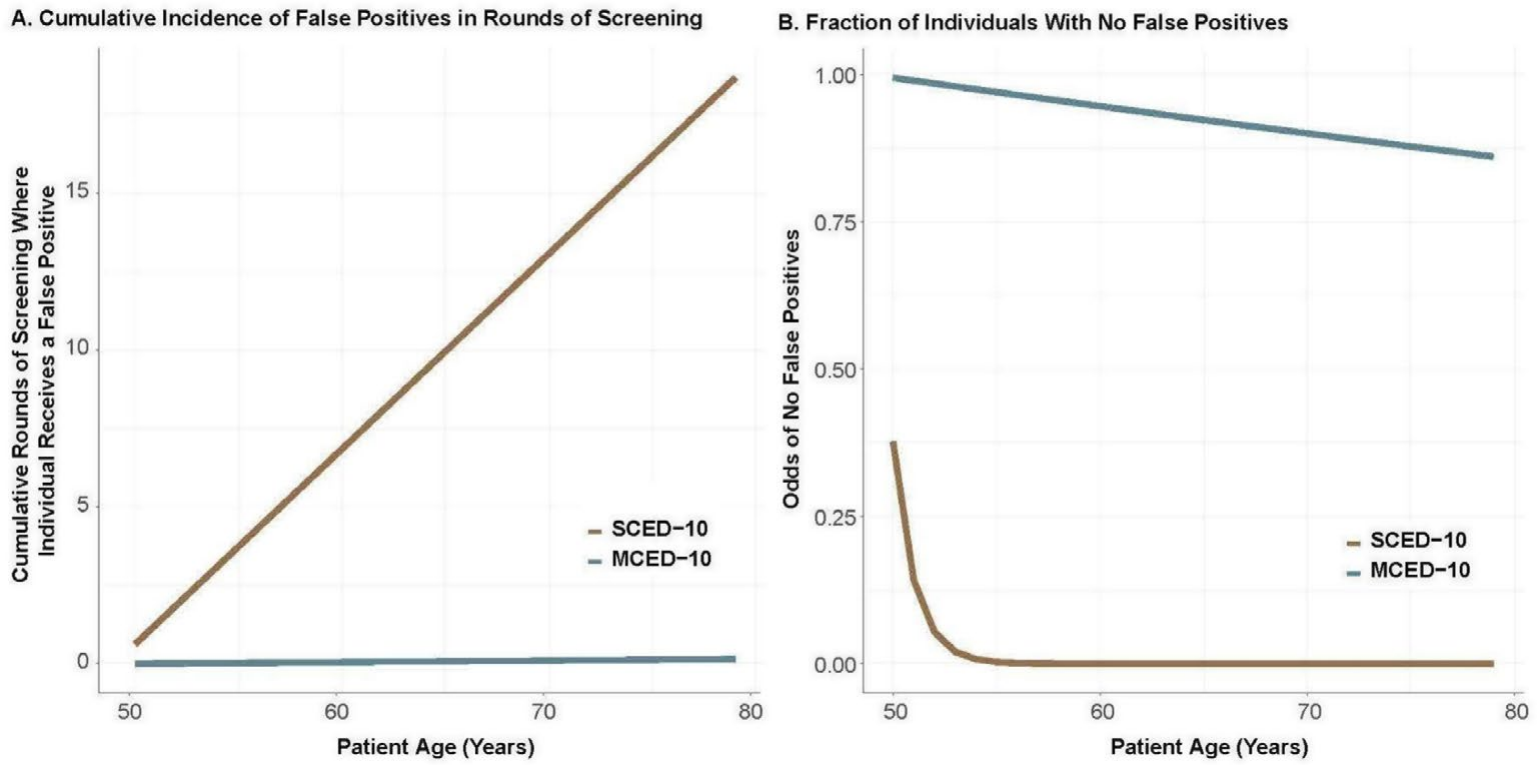
Cumulative incidence of at least one false‐positive result per round of annual screening for each individual from ages 50 to 79 Years under the SCED‐10 and MCED‐10 screening systems. ^a^Assuming 100% adherence and that each individual male receives 7 blood tests and each individual female receives 10 blood tests annually in the hypothetical SCED‐10 system. Abbreviations: MCED, multi‐cancer early detection; SCED, single‐cancer early detection.

The estimated total cost of 1 year of USPSTF guideline‐based screening plus associated diagnostic investigation costs was ~$48 M for 100,000 adults. Estimated incremental total costs for SCED‐10 and MCED‐10 were ~$329 M and $98 M, respectively (Table 4). These results were focused on performance and costs for 1 year of screening. In addition, we estimated the FPR for repeated screening for an individual. Individuals receiving the SCED‐10 screening system annually from ages 50 to 79 years (a duration of 30 years) would be expected to receive false‐positive test results in more than 18 annual rounds of screening per person, on average (Figure 4). This is compared with fewer than 0.12 cumulative false‐positive annual screening rounds per person (30 in total) in the MCED‐10 screening system from ages 50 to 79 years.

**TABLE 4:** Screening costs^a^ of USPSTF, SCED‐10, and MCED‐10 screening systems per 100,000 average‐risk adults aged 50–79 years.

	Reference scenario: USPSTF	Scenario 1: incremental SCED‐10 screen for 10 cancer types	Scenario 2: incremental MCED‐10 screen for 10 cancer types
Investigation costs for false positives	$16,568,945	$242,741,458	$2,179,807
Investigation costs for true positives	$440,534	$1,203,958	$1,320,698
Testing costs	$30,572,233	$84,855,811	$94,739,017
Total costs	$47,581,711	$328,801,227	$98,239,522

Abbreviations: MCED, multi‐cancer early detection; SCED, single‐cancer early detection; USPSTF, United States Preventive Services Task Force.

^a^ Cost calculations apply a 1.5× multiplier to investigation costs for positive MCED results. Investigation costs refer to the cost of cancer investigations in people who are screen‐positive. Testing costs refer to the cost of the screening tests themselves.

Results calculated for the incremental impact of the MCED‐G cancer screening scenario using a commercially available MCED test for more than 50 cancer types are presented in Tables S5–S7 and the Supplemental Results.

## 4 Discussion

Blood‐based SCED and MCED tests represent a new paradigm for cancer detection that can be used in addition to current standard‐of‐care screening. Of course, these tests should first and foremost demonstrate clinical utility and acceptable harm/benefit in randomized controlled trials. However, the availability of such tests has broadened the focus from evaluating blood‐based screening tests one at a time to instead consider the overall strategy of the larger screening system, with the public health goal of limiting harm with acceptable financial costs.

Several blood‐based SCED tests are in development, each targeting a specific cancer type, such as lung, colorectal, bladder, ovarian, pancreatic, or liver [34, 35, 36, 37, 38]. This “one test for one cancer” approach is the same as that traditionally used for population cancer screening since the advent of Pap screening in the mid‐20th century. Here, we demonstrate that if 10 such SCED tests are implemented in a general population, each targeting a different cancer type, this screening system would be inefficient and expensive compared to an analogous MCED approach due to the high number of false‐positive tests requiring diagnostic investigations. Individuals with false‐positive test results would be recalled for imaging and, in many cases, invasive biopsies carrying complication risks, along with associated anxiety, all of which may represent significant harm; for example, longitudinal cohort studies show significant psychological harm that can persist for years among women who test falsely positive for breast cancer [39]. Furthermore, significant evidence supports that false positive testing leads to reduced adherence to future screening, potentially delaying the diagnosis of true cancer [40]. Adherence to multiple SCED tests may also be lower than a single MCED test, which could be exacerbated by individuals who are discouraged from screening because they are confused by multiple test results. This phenomenon may disproportionately harm marginalized groups; evidence supports that African American men with lower educational status were less likely to return for screening follow‐up when faced with a false positive PSA screen [41], and Asian and Hispanic women were less likely to return for screening after a false positive mammogram result [42]. Finally, the PPVs per individual SCED test are low, ranging from 0.10% to 1.20% (summarized in Table S8), as opposed to PPVs in the range of 30%–50% with MCED tests in our analysis. Although no national standard for PPV has been set in the United States, the United Kingdom National Institute for Health and Care Excellence provides a frame of reference for acceptable screening tests, recommending that PPVs exceed 3% [43]. This is important to consider because the individual PPV is a measure of the effectiveness of running such an individual test on an individual, whether or not other tests are also being run.

As shown in our analysis, an MCED test that is limited to target the same 10 cancer types would detect 27% fewer incremental cancers than multiple SCED tests but is more efficient because this screening system would have substantially fewer incremental false positives—approximately 400 for MCED‐10 versus nearly 100,000 for SCED‐10. Because this comparison was based on the same 10 cancer types, it demonstrates clearly that a single MCED test simultaneously covering multiple cancer types is more efficient than implementing multiple SCED tests. This comparison illustrates that a single MCED test with a single FPR, as opposed to multiple FPRs from multiple independent SCED tests, is far more efficient and should be more cost‐effective in a general population setting.

Current USPSTF guideline‐recommended screening covers 4 cancer types that together represent only 15% of all incident cancers in the United States [1, 2]. The 10 cancer types used in our SCED‐10 and MCED‐10 tests, in the screening‐eligible age group of 50 to 79 years, represent 37% of all cancers (54% of those aged 50–79 years) [20]. Given a TPR in the range of 30%–50% [4, 5, 6, 7], the MCED‐10 screening system can maximize the absolute number of incremental cancers found, and coupled with a very low single FPR, represents the most effective and efficient approach for general population screening in our modeling.

We demonstrated that multiple SCED tests cannot feasibly achieve an acceptable overall FPR that is as efficient as a single MCED test (i.e., < 1%). Even though individual tests may each have a relatively high sensitivity, the overall FPR across tests leads to so many potential diagnostic investigations that many healthcare systems would struggle to manage them. This means that ensuring an acceptably low FPR is as important as TPR or sensitivity when considering the overall cancer screening strategy. Our findings are consistent with, for example, the Prostate, Lung, Colorectal, and Ovarian (PLCO) Cancer Screening Trial that demonstrated that an individual receiving multiple single‐cancer screening tests had a ≥ 50% cumulative risk of a false‐positive test result by the end of the 3‐year screening period [44].

The current “one test for one cancer” approach that characterizes standard‐of‐care screening has been and remains effective, but there is limited opportunity to substantially reduce the number of cancer deaths in the general population further, even with highly sensitive SCED tests. For example, within average‐risk populations, an SCED screening approach—even with good risk‐stratification tools and highly efficient individual SCED tests—may have low clinical utility when MCED testing is possible. Furthermore, risk assessment for individual cancer types ignores the fact that a person's risk of all cancers is considerably higher than their risk of any individual cancer; that age is by far the strongest determinant of risk of overall cancer and most individual cancer types; and that other strong risk factors, such as smoking or hereditary cancer syndromes, can be shared across multiple cancer types [45, 46]. Consequently, single‐cancer screening approaches invariably miss the majority of cancers in the general population. As examples, lung cancer comprised only 32% of incident cancers in the National Lung Screening Trial [47], and pancreatic cancer comprised only 11% of incident cancers in the Cancer of Pancreas Screening‐5 (CAPS5) study [48]. Finally, a “single touchpoint” approach to cancer screening across multiple modalities (colonoscopy, mammography, etc.) may promote improved adherence [49], which is particularly relevant for reducing cancer disparities, where screening adherence is a major driver [50].

Some have proposed improving the efficiency and effectiveness of an SCED approach to supplemental screening by targeting SCED testing toward populations that can be risk‐assessed and limited to the highest‐risk groups for a given cancer type, such as for hereditary cancer predisposition disorders [51, 52]. Although this may be feasible with successful risk prediction tools, our findings suggest that this approach would not be feasible in general population settings or across multiple cancer types, including those for which risk factors are poorly understood. We did not compute scenarios in which average‐ and low‐risk individuals for a given cancer were excluded from SCED screening. While it might seem advantageous to focus on test‐level metrics by restricting SCED testing to a small, high‐risk subset of individuals in order to report high PPVs, this approach could overlook program‐level effectiveness by failing to capture cancers in individuals not pre‐identified as high risk. For example, lung cancer screening in high‐risk smoker populations is estimated to exclude one third to more than half of all lung cancer cases [19, 53, 54, 55, 56, 57]. Given that risk factors for other cancers are generally less predictive than smoking is for lung cancer, other strategies for high‐risk targeted screening would most likely exclude even larger proportions of cancer, leading to higher system‐level false‐negative rates. A high‐risk SCED screening approach, in the context of multi‐cancer screening, might therefore be inefficient, as improved detection at the test level would not translate to broad population‐level outcomes. Thus, going forward, tailoring each approach to testing (i.e., SCED vs. MCED) to specific use cases will be important.

Some may argue that examining SCED vs. MCED efficiency prior to proving efficacy (i.e., impact on overall survival) might be “putting the cart before the horse.” At the same time, the sheer scale of false positives and costs inherent in extending the current ‘one test for one cancer’ approach to average‐risk populations emphasizes the need to consider efficiency and resource allocation from the start. This analysis has some limitations related to its goal of comparing hypothetical screening approaches. We did not estimate or incorporate the impacts of screening systems on cancer mortality, overdiagnosis, or stage of detection. We did not calculate the cost‐savings associated with early detection (e.g., less‐aggressive interventions, less treatment, or less‐expensive treatments, all compared to treating advanced‐stage disease, for which there are now many expensive targeted systemic therapies). Furthermore, we did not incorporate the different cancer diagnostic pathways and follow‐up schedules following a positive screening test result for each cancer type. Our modeling included 10 of the cancer types responsible for the most deaths in the United States, which therefore assumed 10 separate tests under the SCED‐10 system. This perhaps represents an extreme scenario, but we aimed to examine screening efficiency in a “best‐case” situation. Additionally, excluding the risks associated with incorrect cancer signal origin results for MCED tests from our analysis likely underestimates the costs of diagnostic investigation associated with a true positive cancer result but an incorrect cancer signal origin prediction from MCED testing. On the other hand, we considered only a single price ($1000) for a hypothetical MCED test; a lower price would result in a correspondingly larger gap in total costs between the MCED and SCED systems. Future research could benefit from more extensive analyses, such as meta‐modeling approaches, to fully capture the impact of changing input parameters and screening strategies [58].

## 5 Conclusion

Our quantitative modeling and analysis demonstrated how a screening system based on a single MCED blood test can simultaneously detect multiple cancer types with high efficiency in a broad population. This is in contrast to the implementation challenges, including higher cost and healthcare utilization, of delivering multiple independent SCED programs in a general population. These results highlight the importance of screening efficiency as part of outcomes analysis and how these practical limitations may impact the practicability of a screening approach regardless of downstream benefits. Our analysis also illustrates how multi‐cancer screening is distinct from a layered set of single cancer tests and has unique advantages as a population screening strategy. Future assessments of novel single‐cancer and multi‐cancer screening approaches should be placed in the context of their potential to reduce the overall burden of cancer in the population.

## Author Contributions

**Sarina Madhavan:** conceptualization (equal), formal analysis (equal), writing – original draft (equal). **Allan Hackshaw:** writing – review and editing (equal). **Earl Hubbell:** conceptualization (equal), data curation (equal), formal analysis (equal), visualization (lead), writing – review and editing (equal). **Ellen T. Chang:** writing – review and editing (equal). **Anuraag Kansal:** data curation (equal), formal analysis (equal), writing – review and editing (equal). **Christina A. Clarke:** conceptualization (equal), supervision (equal), writing – review and editing (equal).

## Conflicts of Interest

S.M. is a former part‐time employee of GRAIL Inc., including during part of the conduct of the study. E.H., E.T.C., A.K., and C.A.C. are current or former employees of GRAIL, report stock or other support from Illumina, and report other support from GRAIL Inc., during the conduct of the study. In addition, EAH has multiple patents in the field of cancer detection pending to GRAIL Inc., A.H. reports an honorarium for an advisory board meeting for GRAIL Inc.; a consultation fee for Evidera for a GRAIL‐initiated project; and previously owned shares in Illumina.

## Supporting information


Data S1.

## Data Availability

All data used in this analysis are publicly available and/or provided in the Supporting Information.
