# Supplementary material for: Estimating the Burden of False Positives and Implementation Costs From Adding Multiple Single Cancer Tests or a Single Multi‐Cancer Test to Standard‐Of‐Care Screening
Source: Cancer Med. 2025 Mar 17;14(6):e70776. doi: 10.1002/cam4.70776 (PMC11912434; doi:10.1002/cam4.70776)
Supplement: Supplementary file 1 — Data S1. [file CAM4-14-e70776-s001.docx]

# Supplemental Materials

Estimating the Burden of False Positives and Implementation Costs From Adding Multiple Single Cancer Tests or a Single Multi-Cancer Test to Standard-of-Care Screening

Sarina Madhavan, MD, MBA,^1,2^Allan Hackshaw, MSc,^3^ Earl Hubbell, PhD,^2^ Ellen T. Chang, ScD,^2,*^ Anuraag Kansal, PhD,^2^ Christina A. Clarke, PhD, MPH^1,2^

1. Massachusetts General Hospital, Boston, Massachusetts, United States of America

2. GRAIL, Inc., Menlo Park, California, United States of America

3. University College of London, London, United Kingdom

*Affiliation at the time of the study.

## Supplemental Table 1. Inputs for Estimates Using Current U.S. Preventive Services Task Force (USPSTF) Screening Guidelines[^1–10^](https://www.zotero.org/google-docs/?GiSVfC)

**​​**

|  | **Modality** | **% Adults Aged 50-79 Years Eligible for Currently**  **Recommended Screening** | **Adherence** | **Screening Interval** | **TPR** | **Specificity (1 - False-Positive Rate)** |
| --- | --- | --- | --- | --- | --- | --- |
| Breast | Mammography | 90% of women | 78% | Annually^d^ | 87% | 89% |
| Colorectal | Cologuard^c^ | 93% | 70% | Every 3 years | 92% | 87% |
| Cervical | Cotesting^a^ | 62% of women | 80% | Every 5 years | 95% | 85% |
| Lung | LDCT | 15%^b^ | 7% | Annually | 85% | 87% |

^a^ Cotesting = Cervical cytology and high-risk human papillomavirus (hrHPV) testing together. The Behavioral Risk Factor Surveillance System surveys for Pap testing alone rather than cotesting, as many women are unsure about whether they have received hrHPV cotesting.[^11^](https://www.zotero.org/google-docs/?Ztfw3d) As a result, there is no reliable estimate of cotesting uptake.

^b^Approximately 69% of lung cancer patients are eligible for currently recommended screening based on high-risk smoking criteria.[^7^](https://www.zotero.org/google-docs/?0X19lI)

^c^ Cologuard is a multi-target stool DNA test. We chose these performance characteristics and screening interval because Cologuard is most comparable to liquid biopsy screening paradigms given that it is non-interventional and has reported performance criteria (unlike fecal immunochemical testing).

^d^Although the USPSTF recommends biennial screening, the American Cancer Society and National Comprehensive Cancer Network recommend annual screening for some age groups. We assume annual screening in order to assume maximum benefits associated with current screening.

LDCT, Low-dose computed tomography; TPR, true positive rate.

## Supplemental Table 2: Key Assumptions for Estimating the Performance Efficiency of SCED-10 and MCED-10 Screening Systems. Additional assumptions are presented in the Supplemental Materials, including Supplemental Tables 1 and 3.

| ***USPSTF Screening*** |
| --- |
| We did not impute an average TPR of the USPSTF system given the differences in how TPR is calculated across each test |
| When calculating performance including TPR and FPR of each test, we used the upper end of the literature-supported range for each in order to increase the number of cancers detected by current screening, and thus reduce the number available to be detected by supplemental screening |
| When calculating adherence, we used studies with higher estimates of screening uptake and assumed an annual frequency of mammography (based on American Cancer Society guidelines, as opposed to biennial, based on USPSTF guidelines) in order to increase the number of cancers detected by current screening, and thus reduce the number available to be detected by supplemental screening |
| ***SCED-10*** |
| We did not impute an average TPR of the USPSTF system given the differences in how TPR is calculated across each test |
| The TPRs and FPR of each hypothetical SCED test in the SCED-10 system is that of mammography (TPR [87%] and FPR [11%]) given that this is a widely accepted and guideline recommended cancer screening test and has a FPR in the midway range for approved cancer screening tests |
| ***MCED-10*** |
| All individuals receive the same MCED test, whereas men and women receive different sets of tests under SCED-10 and under currently recommended screening guidelines, as some screening tests are sex-specific. |
| The TPR and FPR of the hypothetical MCED-10 test/system is modeled after Klein et al, 2021 but the TPRs (cancer and stage-specific) is falsely restricted to the same 10 cancer types as SCED-10 for sake of comparison.[^12^](https://www.zotero.org/google-docs/?scDirv) The FPR is the same as in Klein et al. (0.4%)[^12^](https://www.zotero.org/google-docs/?mctAn0) |
| False positives calculated are distributed across cancer types in order to approximate investigation costs |
| ***Cost analysis*** |
| Cost calculations apply a 1.5 multiplier to the costs of cancer investigations following positive MCED test results to more accurately compare competing risks and reflect real-world cost variability. |

FPR, false positive rate; MCED, multi-cancer early detection; SCED, single-cancer early detection; TPR, true positive rate; USPSTF; United States Preventive Services Task Force.

## Supplemental Table 3. Sensitivities of an MCED Test by Cancer Site and Stage (Detected Cancer/Total Cancer), Including Observed^a^ and Modified to be Non-Decreasing Across Stages By Applying Weighted Isotonic Regression

| **Cancer Site** | **Clinical Stage** | **Total** | **Test Positive** | **TPR (95% CI)** | **Isotonic TPR** |
| --- | --- | --- | --- | --- | --- |
| Overall | All | 2823 | 1453 | 51.5% (49.6-53.3%) | 51.5% |
| Anus | All | 22 | 18 | 81.8% (61.5-92.7%) | 81.8% |
|  | I | 4 | 1 | 25.0% (1.3-69.9%) | 25.0% |
|  | II | 4 | 3 | 75.0% (30.1-98.7%) | 75.0% |
|  | III | 13 | 13 | 100.0% (77.2-100.0%) | 100.0% |
|  | IV | 1 | 1 | 100.0% (5.1-100.0%) | 100.0% |
| Bladder | All | 23 | 8 | 34.8% (18.8-55.1%) | 34.8% |
|  | I | 6 | 2 | 33.3% (9.7-70.0%) | 17.6% |
|  | II | 11 | 1 | 9.1% (0.5-37.7%) | 17.6% |
|  | III | 4 | 3 | 75.0% (30.1-98.7%) | 75.0% |
|  | IV | 2 | 2 | 100.0% (34.2-100.0%) | 100.0% |
| Breast | All | 524 | 160 | 30.5% (26.7-34.6%) | 30.5% |
|  | I | 265 | 7 | 2.6% (1.3-5.4%) | 2.6% |
|  | II | 181 | 86 | 47.5% (40.4-54.8%) | 47.5% |
|  | III | 55 | 47 | 85.5% (73.8-92.4%) | 85.5% |
|  | IV | 22 | 20 | 90.9% (72.2-97.5%) | 90.9% |
|  | Missing | 1 | 0 | 0.0% (0.0-94.9%) | – |
| Cervix | All | 25 | 20 | 80.0% (60.9-91.1%) | 80.0% |
|  | I | 12 | 7 | 58.3% (32.0-80.7%) | 58.3% |
|  | II | 5 | 5 | 100.0% (56.6-100.0%) | 100.0% |
|  | III | 7 | 7 | 100.0% (64.6-100.0%) | 100.0% |
|  | IV | 1 | 1 | 100.0% (5.1-100.0%) | 100.0% |
| Colon/Rectum | All | 206 | 169 | 82.0% (76.2-86.7%) | 82.0% |
|  | I | 30 | 13 | 43.3% (27.4-60.8%) | 43.3% |
|  | II | 40 | 34 | 85.0% (70.9-92.9%) | 85.0% |
|  | III | 66 | 58 | 87.9% (77.9-93.7%) | 87.9% |
|  | IV | 64 | 61 | 95.3% (87.1-98.4%) | 95.3% |
|  | Missing | 6 | 3 | 50.0% (18.8-81.2%) | – |
| Esophagus | All | 100 | 85 | 85.0% (76.7-90.7%) | 85.0% |
|  | I | 8 | 1 | 12.5% (0.6-47.1%) | 12.5% |
|  | II | 17 | 11 | 64.7% (41.3-82.7%) | 64.7% |
|  | III | 34 | 32 | 94.1% (80.9-98.4%) | 94.1% |
|  | IV | 40 | 40 | 100.0% (91.2-100.0%) | 100.0% |
|  | Missing | 1 | 1 | 100.0% (5.1-100.0%) | – |
| Gallbladder | All | 17 | 12 | 70.6% (46.9-86.7%) | 70.6% |
|  | I | 2 | 0 | 0.0% (0.0-65.8%) | 0.0% |
|  | II | 3 | 1 | 33.3% (1.7-79.2%) | 33.3% |
|  | III | 4 | 3 | 75.0% (30.1-98.7%) | 75.0% |
|  | IV | 8 | 8 | 100.0% (67.6-100.0%) | 100.0% |
| Head and Neck | All | 105 | 90 | 85.7% (77.8-91.1%) | 85.7% |
|  | I | 19 | 12 | 63.2% (41.0-80.9%) | 63.2% |
|  | II | 17 | 14 | 82.4% (59.0-93.8%) | 82.4% |
|  | III | 19 | 16 | 84.2% (62.4-94.5%) | 84.2% |
|  | IV | 50 | 48 | 96.0% (86.5-98.9%) | 96.0% |
| Kidney | All | 99 | 18 | 18.2% (11.8-26.9%) | 18.2% |
|  | I | 61 | 3 | 4.9% (1.7-13.5%) | 4.9% |
|  | II | 9 | 2 | 22.2% (6.3-54.7%) | 18.8% |
|  | III | 7 | 1 | 14.3% (0.7-51.3%) | 18.8% |
|  | IV | 22 | 12 | 54.5% (34.7-73.1%) | 54.5% |
| Liver/Bile-duct | All | 46 | 43 | 93.5% (82.5-97.8%) | 93.5% |
|  | I | 6 | 6 | 100.0% (61.0-100.0%) | 81.3% |
|  | II | 10 | 7 | 70.0% (39.7-89.2%) | 81.3% |
|  | III | 9 | 9 | 100.0% (70.1-100.0%) | 100.0% |
|  | IV | 20 | 20 | 100.0% (83.9-100.0%) | 100.0% |
|  | Missing | 1 | 1 | 100.0% (5.1-100.0%) | – |
| Lung | All | 404 | 302 | 74.8% (70.3-78.7%) | 74.8% |
|  | I | 96 | 21 | 21.9% (14.8-31.1%) | 21.9% |
|  | II | 44 | 35 | 79.5% (65.5-88.8%) | 79.5% |
|  | III | 118 | 107 | 90.7% (84.1-94.7%) | 90.7% |
|  | IV | 145 | 138 | 95.2% (90.4-97.6%) | 95.2% |
|  | Missing | 1 | 1 | 100.0% (5.1-100.0%) | – |
| Lymphoid Leukemia^b^ | All | 51 | 21 | 41.2% (28.8-54.8%) | 41.2% |
| Lymphoma | All | 174 | 98 | 56.3% (48.9-63.5%) | 56.3% |
|  | I | 33 | 9 | 27.3% (15.1-44.2%) | 27.3% |
|  | II | 48 | 28 | 58.3% (44.3-71.2%) | 58.3% |
|  | III | 46 | 33 | 71.7% (57.5-82.7%) | 66.3% |
|  | IV | 46 | 28 | 60.9% (46.5-73.6%) | 66.3% |
|  | Missing | 1 | 0 | 0.0% (0.0-94.9%) | – |
| Melanoma | All | 13 | 6 | 46.2% (23.2-70.9%) | 46.2% |
|  | I | 2 | 0 | 0.0% (0.0-65.8%) | 0.0% |
|  | II | 2 | 0 | 0.0% (0.0-65.8%) | 0.0% |
|  | III | 3 | 0 | 0.0% (0.0-56.1%) | 0.0% |
|  | IV | 6 | 6 | 100.0% (61.0-100.0%) | 100.0% |
| Myeloid Neoplasm^b^ | All | 10 | 2 | 20.0% (5.7-51.0%) | 20.0% |
| Ovary | All | 65 | 54 | 83.1% (72.2-90.3%) | 83.1% |
|  | I | 10 | 5 | 50.0% (23.7-76.3%) | 50.0% |
|  | II | 5 | 4 | 80.0% (37.6-99.0%) | 80.0% |
|  | III | 31 | 27 | 87.1% (71.1-94.9%) | 87.1% |
|  | IV | 19 | 18 | 94.7% (75.4-99.7%) | 94.7% |
| Pancreas | All | 135 | 113 | 83.7% (76.6-89.0%) | 83.7% |
|  | I | 21 | 13 | 61.9% (40.9-79.2%) | 61.0% |
|  | II | 20 | 12 | 60.0% (38.7-78.1%) | 61.0% |
|  | III | 21 | 18 | 85.7% (65.4-95.0%) | 85.7% |
|  | IV | 73 | 70 | 95.9% (88.6-98.6%) | 95.9% |
| Plasma Cell Neoplasm^b^ | All | 47 | 34 | 72.3% (58.2-83.1%) | 72.3% |
| Prostate | All | 420 | 47 | 11.2% (8.5-14.6%) | 11.2% |
|  | I | 95 | 3 | 3.2% (1.1-8.9%) | 3.2% |
|  | II | 243 | 12 | 4.9% (2.8-8.4%) | 4.9% |
|  | III | 50 | 7 | 14.0% (7.0-26.2%) | 14.0% |
|  | IV | 30 | 25 | 83.3% (66.4-92.7%) | 83.3% |
|  | Missing | 2 | 0 | 0.0% (0.0-65.8%) | – |
| Sarcoma | All | 30 | 18 | 60.0% (42.3-75.4%) | 60.0% |
|  | I | 10 | 4 | 40.0% (16.8-68.7%) | 40.0% |
|  | II | 2 | 2 | 100.0% (34.2-100.0%) | 58.3% |
|  | III | 10 | 5 | 50.0% (23.7-76.3%) | 58.3% |
|  | IV | 7 | 6 | 85.7% (48.7-99.3%) | 85.7% |
|  | Missing | 1 | 1 | 100.0% (5.1-100.0%) | – |
| Stomach | All | 30 | 20 | 66.7% (48.8-80.8%) | 66.7% |
|  | I | 6 | 1 | 16.7% (0.9-56.4%) | 16.7% |
|  | II | 6 | 3 | 50.0% (18.8-81.2%) | 50.0% |
|  | III | 5 | 4 | 80.0% (37.6-99.0%) | 80.0% |
|  | IV | 12 | 12 | 100.0% (75.8-100.0%) | 100.0% |
|  | Missing | 1 | 0 | 0.0% (0.0-94.9%) | – |
| Thyroid | All | 14 | 0 | 0.0% (0.0-21.5%) | 0.0% |
|  | I | 11 | 0 | 0.0% (0.0-25.9%) | 0.0% |
|  | II | 1 | 0 | 0.0% (0.0-94.9%) | 0.0% |
|  | III | 1 | 0 | 0.0% (0.0-94.9%) | 0.0% |
|  | IV | 1 | 0 | 0.0% (0.0-94.9%) | 0.0% |
| Urothelial Tract | All | 10 | 8 | 80.0% (49.0-94.3%) | 80.0% |
|  | I | 2 | 0 | 0.0% (0.0-65.8%) | 0.0% |
|  | II | 0 | 0 | – | 0.0% |
|  | III | 0 | 0 | – | 0.0% |
|  | IV | 8 | 8 | 100.0% (67.6-100.0%) | 100.0% |
| Uterus | All | 157 | 44 | 28.0% (21.6-35.5%) | 28.0% |
|  | I | 120 | 20 | 16.7% (11.1-24.3%) | 16.7% |
|  | II | 10 | 3 | 30.0% (10.8-60.3%) | 30.0% |
|  | III | 23 | 17 | 73.9% (53.5-87.5%) | 73.9% |
|  | IV | 4 | 4 | 100.0% (51.0-100.0%) | 100.0% |
| Other^c^ | All | 59 | 30 | 50.8% (38.4-63.2%) | 50.8% |
|  | I | 11 | 2 | 18.2% (5.1-47.7%) | 0.0% |
|  | II | 3 | 3 | 100.0% (43.9-100.0%) | 41.2% |
|  | III | 18 | 13 | 72.2% (49.1-87.5%) | 20.0% |
|  | IV | 18 | 11 | 61.1% (38.6-79.7%) | 72.3% |
|  | Missing | 3 | 1 | 33.3% (1.7-79.2%) | – |

Two-sided 95% Wilson confidence intervals were calculated.

For multiple primaries, the highest clinical stage was selected.

^a^Reproduced from Klein et al., Table S5.[^12^](https://www.zotero.org/google-docs/?3pHRsV)

^b^Not expected to be staged.

^c^The Other cancer class includes: adrenal (*n*=1), ampulla of vater (*n*=1), brain (*n*=6), choriocarcinoma (*n*=1), mesothelioma (*n*=7), non-melanoma non-BCC/SCC skin cancer (*n*=2), other/unspecified (*n*=10), penis (*n*=1), small intestine (*n*=13), testis (*n*=6), thymus (*n*=2), vagina (*n*=2), vulva (*n*=7).

BCC, basal cell carcinoma; CI, confidence interval; MCED, multi-cancer early detection; SCC, squamous cell carcinoma.

## Supplemental Table 4. Summary of Diagnostic Work-up Cost for US Following a Positive Screening Test Result[^1^](https://www.zotero.org/google-docs/?dx75lr)

| **Cancer** | **Procedure*** | **ICD-10 Code** | **Additional Procedures** | **CPT Code** | **Total** |
| --- | --- | --- | --- | --- | --- |
| Lung and Bronchus | LDCT for lung ca screen | G0297 | Biopsy, lung or mediastinum, percutaneous needle | 32405 | $3,730.99 |
| Colon and Rectum | Colonoscopy, flexible; diagnostic, including collection of specimen(s) by brushing or washing, when performed (separate procedure) | 45378 | NA | ·· | $799.78 |
| Lymphoma | CT Chest w/ Contrast | 71260 | Biopsy or excision of lymph node(s), by needle, superficial | 38505 | $3,593.96 |
| Breast Cancer | Mammogram Bilateral | 77066 | Biopsy of breast, percutaneous, needle core, not using imaging guidance | 19100 | $3,569.97 |
| Pancreas | CT Abd W W/O Contrast | 74170 | Transendoscopic ultrasound-guided intramural or transmural fine-needle aspiration/biopsy(s) Esophagogastroduodenoscopy, flexible, transoral | 43242 | $4,019.00 |
| Head and Neck | E&M Moderate to High Severity (40 minutes face-to-face) | 99215 | Diagnostic laryngoscopy | 31575 | $719.10 |
| Liver and Intrahepatic Bile Duct | US Abdomen Complete | 76700 | Biopsy of liver, needle, percutaneous | 47000 | $3,462.88 |
| Ovary | Ca screen;pelvic/breast exam | G0101 | US Abdomen Complete | 76700 | $399.69 |
| Stomach | Esophagogastroduodenoscopy, flexible, transoral; diagnostic, including collection of specimen(s) by brushing or washing, when performed | 43235 | NA | ·· | $274.37 |
| Esophagus | Esophagogastroduodenoscopy, flexible, transoral; diagnostic, including collection of specimen(s) by brushing or washing, when performed | 43235 | NA | ·· | $274.37 |
| Kidney | CT Abd & Pelvis W W/O Contrast | 74178 | Renal biopsy; percutaneous, by trocar or needle | 50200 | $4,053.11 |
| Prostate | MRI Pelvis W W/O Contrast | 72197 | Biopsy, prostate; needle or punch, single or multiple, any approach | 55700 | $4,970.69 |
| Multiple Myeloma | Comprehensive (13 Tests - Multiple Codes) | Multiple Codes | NA | ·· | $158.93 |
| Leukemia | Bl smear w/diff wbc count | 85007 | NA | ·· | $26.61 |
| Indeterminate | CT Extremity Upper W/ Contrast | 73201 | NA | ·· | $896.13 |
| Uterine | Ca screen;pelvic/breast exam | G0101 | US Abdomen Complete | 76700 | $399.69 |
| Bladder | CT Abd & Pelvis W W/O Contrast | 74178 | Renal biopsy; percutaneous, by trocar or needle | 50200 | $4,053.11 |
| Melanoma | Punch Skin Biopsy Single Lesion | 11104 | NA | ·· | $314.20 |
| Cervical | Ca screen;pelvic/breast exam | G0101 | NA | ·· | $111.67 |
| Anal | Colonoscopy, flexible; diagnostic, including collection of specimen(s) by brushing or washing, when performed (separate procedure) | 45378 | NA | ·· | $799.78 |

*Note: All workups were assumed to begin with a complete blood count (HCPCS code 85025); commercial costs were assumed to be 2.3x Medicare rate. All workup costs related to MCED testing (MCED-10 and MCED-G) were assumed to be 1.5x that of SCED testing workup due to assumed additional complexity.

ICD, International Classification of Diseases; W, with; W/O, without; CT, computed tomography.

## Supplemental Table 5: Cancers Detected, Diagnostic Investigations in People Without Cancer, and Ratio per Year for USPSTF, SCED-10, MCED-10, and MCED-G Screening Systems Among 100,000 Average-risk^a^ Adults Aged 50-79 Years

|  | **Reference Scenario:** USPSTF | Scenario 1: Incremental SCED-10 Screen for 10 Cancer Types | Scenario 2: Incremental MCED-10 Screen for 10 Cancer Types | Scenario 3: Incremental MCED-G Screen for >50 Cancer Types |
| --- | --- | --- | --- | --- |
| Total # of Cancers Eligible | 356 | 473 | 473 | 1,013 |
| Total # of Tests Performed | 62,524 | 848,558 | 99,830 | 99,830 |
| Cancers Detected (% of Total Cancers Eligible) | 170 (48%) | 412 (87%) | 298 (63%) | 426 (42%) |
| Diagnostic Investigations in People Without Cancer (% of Total Tests) | 7,496 (12%) | 93,289 (11%) | 497 (0.5%) | 494 (0.5%) |
| Ratio of Cancers Detected to Diagnostic Investigations in People Without Cancer | 0.02 | 0.004 | 0.60 | 0.86 |
| People Without Cancer Receiving Diagnostic Investigations (% of Population) | 7,304 (7.3%) | 62,167 (62.2%) | 497 (0.5%) | 494 (0.5%) |

^a^In this context, “average-risk” includes all adults aged 50-79 years and those for whom USPSTF recommends low-dose computed tomography screening for lung cancer, i.e., adults in this age group who have a 20 pack-year smoking history and currently smoke or have quit within the past 15 years.

MCED, multi-cancer early detection; SCED, single-cancer early detection; USPSTF, United States Preventive Services Task Force.

## Supplemental Table 6: Efficiency Metrics of USPSTF, SCED-10, MCED-10, and MCED-G Screening Systems in 100,000 Average-risk Adults Aged 50-79 Years

|  | **Reference Scenario:** USPSTF | **Scenario 1**: Incremental SCED-10 Screen for 10 Cancer Types | **Scenario 2**: Incremental MCED-10 Screen for 10 Cancer Types | **Scenario 3**: Incremental MCED-G Screen for >50 Cancer Types |
| --- | --- | --- | --- | --- |
| Positive Predictive Value | 2.21% | 0.44% | 37.53% | 46.30% |
| Aggregate Negative Predictive Value (for Targeted Cancer Types) | 99.8% | 99.9% | 99.8% | 99.4% |
| All-Cancer Negative Predictive Value (for All Cancer Types) | 99.0% | 99.4% | 99.3% | 99.4% |
| Number Needed to Screen | 369 | 2,062 | 334 | 234 |

MCED, multi-cancer early detection; SCED, single-cancer early detection; USPSTF, United States Preventive Services Task Force.

## Supplemental Table 7: Screening Costs^a^ of USPSTF, SCED-10, MCED-10, and MCED-G Screening Systems per 100,000 Average-risk Adults Aged 50-79 Years

|  | **Reference Scenario:** USPSTF | **Scenario 1:** Incremental SCED-10 Screen for 10 Cancer Types | **Scenario 2:** Incremental MCED-10 Screen for 10 Cancer Types | **Scenario 3:** Incremental MCED-G Screen for >50 Cancer Types |
| --- | --- | --- | --- | --- |
| Investigation Costs for False Positives | $16,568,945 | $242,741,458 | $2,179,807 | $2,105,944 |
| Investigation Costs for True Positives | $440,534 | $1,203,958 | $1,320,698 | $1,659,001 |
| Testing Costs | $30,572,233 | $84,855,811 | $94,739,017 | $94,739,017 |
| Total Costs | $47,581,711 | $328,801,227 | $98,239,522 | $98,503,962 |

^a^Cost calculations apply a 1.5x multiplier to investigation costs for positive MCED results. Investigation costs refer to the cost of cancer investigations in people who are screen-positive. Testing costs refer to the cost of the screening tests themselves.

MCED, multi-cancer early detection; SCED, single-cancer early detection; USPSTF, United States Preventive Services Task Force.

## Supplemental Table 8. Positive Predictive Values (PPVs) of Each Hypothetical SCED Test Within the SCED-10 System for Individual Cancer Subtypes

| Cancer Type | PPV per Hypothetical SCED test |
| --- | --- |
| Lung and Bronchus | 1.21% |
| Colon and Rectum | 0.37% |
| Pancreas | 0.25% |
| Liver and Intrahepatic Bile Duct | 0.21% |
| Breast | 1.09% |
| Lymphoma | 0.39% |
| Esophagus | 0.10% |
| Ovary | 0.30% |
| Uterine Corpus | 0.62% |
| Bladder | 0.19% |

CRC, Breast PPVs are low for supplemental SCED testing in this modeling in part due to the fact that many of the potential cancers have been previously screened out by USPSTF screening – thus reducing the pretest probability of a true positive for supplemental screening.

CRC, colorectal cancer; SCED, single-cancer early detection; USPSTF, United.States Preventive Services Task Force.

# Supplemental Methods

*Cancer incidence data*

Cancer incidence rates for the US general population aged 50-79 years were obtained from the Surveillance, Epidemiology, and End Results registries for 17 geographic regions in 2006-2015, based on data released in April 2022.[^13^](https://www.zotero.org/google-docs/?VRFtOm) These diagnosis years were chosen to enable uniform classification of all patients according to the 6th edition of the American Joint Committee on Cancer staging system.

*Current guideline-recommended screening parameters*

**Supplemental Table 1** summarizes model input parameters. USPSTF guidelines recommend biennial breast cancer screening using mammography for women aged 50-79 years, while draft USPSTF guidelines recommend biennial screening for women aged 40-79 years.[^14^](https://www.zotero.org/google-docs/?HEk5jp) For purposes of this study, we assume annual screening for breast cancer using mammography among women aged 50-79 years, annual lung cancer screening using low-dose computed tomography (LDCT) for adults aged 55-80 years who have a 20-pack-year smoking history and currently smoke or have quit within the past 15 years, cervical cancer screening every 5 years using cytology and high-risk human papillomavirus (hrHPV) testing for women aged 50-65 years, and colorectal cancer screening using a fecal DNA test every 3 years for adults aged 50-75 years.[^2–6^](https://www.zotero.org/google-docs/?y7NRGb) Nationally representative estimates of uptake came from the Behavioral Risk Factor Surveillance System (BRFSS).[^9^](https://www.zotero.org/google-docs/?IDmvOk)

*Screening performance for currently recommended screening*

We used the published performance (TPR and false positive rate [FPR]) for each of the four USPSTF-recommended tests.[^2–6^](https://www.zotero.org/google-docs/?4QvxCw) TPR (also called sensitivity) was defined as the proportion of people with cancer who receive a positive test result; FPR (also called 1 minus specificity) was defined as the proportion of people without cancer who receive a positive test result. Test TPR (test sensitivity) reflects the ability of a screening test to detect cancer when the person’s cancer status at the time of test is known. This can be in a clinically diagnosed cohort or in a screening setting when a definitive diagnostic test is undertaken by all study participants. Episode TPR (episode sensitivity) reflects the ability of a screening test to detect cancers that would be diagnosed during a follow-up period after a specific round of a screening test; this depends on the natural history of the cancer and the defined interval of screening (e.g., one year). We use a simplified model in this paper and assume that screening intervals are sufficient for test TPR and episode TPR to be similar.

*Screening performance for single-cancer early detection (SCED) system*

We assumed 100% uptake for each of 10 hypothetical SCED tests with the performance (TPR and FPR) of mammography.[^6^](https://www.zotero.org/google-docs/?Q2oX7J)

*Screening performance for multi-cancer early detection (MCED) systems (MCED-10 and MCED-G)*

MCED-10 and MCED-G were hypothetical tests designed using performance characteristics of the Galleri^®^ blood test, which utilizes targeted methylation analysis of circulating cell-free DNA (cfDNA) to simultaneously detect multiple cancer types.[^12,15^](https://www.zotero.org/google-docs/?awQTBI) In the hypothetical scenario labeled “MCED-10”, we measured the impact of a single MCED test per person targeting 10 cancer types. In order to reflect MCED technology as intended, we also calculated the incremental impact of a third scenario using the performance of the Galleri^®^ MCED test for >50 types of cancer (labeled “MCED-G”). We assumed 100% uptake for each MCED blood test to reflect maximum possible gains as well as maximum possible diagnostic costs. Galleri^®^ test TPR varies by cancer type and stage and was determined by the proportion of tumors that shed detectable amounts of cfDNA; this is summarized in **Supplemental Table 2**. Detection of shed cfDNA was assumed to be generally reliable when above the limit of detection. The average TPR (calculated as a weighted average) was 52% for MCED-G and 59% for MCED-10, and the FPR for both tests was 0.4%.[^15^](https://www.zotero.org/google-docs/?6OiW4L) An isotonic regression, using the pool adjacent violators algorithm in the software R, was applied to ensure TPR was non-decreasing with increasing stage within a specific cancer type.[^16^](https://www.zotero.org/google-docs/?FVEiKz) The performance results of MCED tests should be interpreted with caution because they were derived from a case-control study,[^12^](https://www.zotero.org/google-docs/?f0WJ8E) which may not accurately reflect the actual effectiveness of MCED tests in real-world clinical settings. In contrast, the performance results of single cancer screening tests were based on data obtained from clinical practice or randomized clinical trials.

All calculations presented in this context estimate incident screening rounds, which are in contrast to a prevalent screening round, represent typical years within an ongoing screening program. The prevalent screening round, or the first year in which screening is introduced to a population is well-understood to cause a transient increase in cancer as it “pulls forward” cancers that would have been diagnosed in subsequent years in the absence of screening. The number of cancers detected in a prevalent screen is influenced by the uncertain rate at which previously undetected cancers progress to a detectable stage. In contrast, incident screening rounds reflect a steady-state scenario, where the total incidence of cancer in subsequent years of an ongoing screening program equals the total incidence in the absence of the program. This equilibrium is achieved by balancing cancers pulled forward from the current year to earlier years with cancers pulled from future years to the current year, assuming for simplicity, no overdiagnosis. Consequently, the number of cancers detected by screening in an incident screen remains unaffected by lead-time bias. However, both earlier average stage at diagnosis and related clinical benefits can still be influenced. It is important to acknowledge that screening may lead to some overdiagnosis, which is difficult to estimate quantitatively.

*Calculating TPs and FPs*

For a single cancer, the number of true positives (TPs) was calculated as follows:

**adherence × eligible population × TPR × overall incidence**

Adherence was defined as the percentage of the population adherent to guidelines for that screening test; eligible population is defined as the percentage of the 50- to 79-year-old adult population qualifying for screening according to USPSTF A and B recommendations[^2^](https://www.zotero.org/google-docs/?ZGEYj4); TPR was the TPR of the test for invasive cancer (i.e., not including precancerous lesions); and overall incidence was the incidence across all stages of the cancer type being screened for.

The number false positives (FPs) in the target age group (50-79 years in the US) identified by the recommended screening tests for a one-year period were calculated as follows:

**proportion of eligible cancers screened × screening interval × adherence × TPR × FPR**.

We applied these calculations to the cancers with USPSTF A and B recommendation criteria. The proportion of cancers potentially screened was defined as the percent of cancers that meet eligibility criteria (e.g., 90% of breast cancers in women aged 50-79 years meet the eligibility criteria for recommended screening, since the maximum recommended age is 74 years). We assumed that approximately 69% of patients with lung cancer aged 50-79 years meet the eligibility criteria for current screening based on smoking criteria,[^7^](https://www.zotero.org/google-docs/?uSlGzl) although this figure may be an overestimate, which would in turn lead to underestimation of the incremental impact of SCED-10, MCED-10, or MCED-G screening.

While it is possible for an individual to undergo screening within multiple screening programs, we considered each screening test as a separate entity in order to encompass all potential diagnostic costs. Additionally, for purposes of simplicity, we assumed that no individual included in the incident screen had two distinct primary cancers.

For the SCED-10, and MCED-10, and MCED-G modeling, we calculated the TPs of each of the hypothetical screening systems as incremental to USPSTF screening. Thus, we excluded cancers detected by USPSTF screening from the pool of cancers potentially detected by these supplemental screening systems as follows:

**Uptake × [TPR x (Incidence - Cancers detected by USPSTF screening)]**.

We assumed that uptake was 100% for each of the supplemental screening systems to maximize the potential cancers detected and costs associated with testing. For the SCED-10 system, we calculated TPs for each of the 10 cancers assuming the TPR of each test was 89% (i.e., the same as that of mammography).

The calculation for MCED-10 and MCED-G was stage-specific, given that the sensitivities reported by MCED tests are reported by stage. Thus, for a single cancer type, the number of TPs for the MCED test was calculated as follows:

**MCED uptake × S_stages_ [MCED TPR_stage_ × (incidence_stage_-cancers detected by single cancer screening)]**.

The calculation involves summing the numbers of invasive cancer stages (stages I - IV) and includes various factors. These factors include MCED uptake, which is the product of the number of adults in the target population and the assumed adherence rate; MCED TPR_stage_, representing the TPR of the MCED test for each cancer stage; incidence_stage_, indicating the incidence of a specific cancer type for each stage; and cancers detected by single cancer screening, which was calculated similarly for cancers with recommended screenings and set to 0 for those without. The total number of cancers detected by MCED was then obtained by summing the detected cancers for each cancer type.

This calculation provides an estimate of the number of cases that would be detected by MCED according to the stage at clinical presentation. However, it does not determine the stage at which MCED detects these cancers. Since it does not predict the stage at detection, this calculation relies solely on the observed stage distribution of incident cancers.

These calculations were based on several assumptions. We assumed that there were no interval cancers with current single cancer screenings, meaning that all observed cancers were available for detection. This assumption introduces a slight bias, increasing the number of cancers detected by single cancer screenings and reducing the number of cancers potentially detectable by MCED.

Additionally, we assumed that single cancer screenings detect the same fraction of observed incident cancers at all stages. In practice, it is likely that current screenings detect a higher proportion of early-stage cancer diagnoses compared to late-stage diagnoses among cancers with available screenings. This simplifying assumption results in a relatively higher number of early-stage cancers and a relatively lower number of late-stage cancers available for detection by MCED. Since MCED TPR increases with the stage, this assumption introduces a modest bias that reduces the number of cancers detected by MCED.

Furthermore, these calculations assume no overdiagnosis. We accounted only for cancers that would have occurred in the absence of MCED testing, thereby underestimating the number of cancers detected by MCED. The potential harms associated with overdiagnosed cancers should be considered separately and are discussed in the limitations of this analysis.

The number of FPs for each supplemental screening system was computed as follows:

**((uptake – individuals with cancer detected) × FPR) / screening interval**

We assumed that those individuals with cancer detected included only those within the fraction adherent to screening. The screening interval for annual screening would be 1, as we assumed for each of the supplemental screening systems.

The analyses produced the number of additional cancers (all cancer types) that could be detected by any of the supplemental systems (SCED-10, MCED-10, or MCED-G) during a one-year period, distinct from those detected through USPSTF-recommended screening. Additional cancers found by an MCED blood test include breast, cervix, colorectal, and lung cancers among people aged 50-79 years who were not eligible for current screening based on existing criteria, as well as those among eligible people who were screened but had an existing cancer that was missed by the test. The additional number of FPs was determined by applying the 0.4% FPR to the target population size (minus the number of cancers detected by recommended screening).

*Three supplemental screening scenarios*

This study focused on a target population of adults aged 50-79 years in the United States Three different scenarios were considered, as explained in Figure 2 of the manuscript. The base case scenario assumed that eligible individuals would undergo recommended screening tests within the specified age range for those tests. The first supplemental screening scenario, called SCED-10, involved a system of blood tests known as single-cancer early detection (SCED) for ten different cancers. These tests were administered to individuals who had previously tested negative in the currently recommended tests, as well as those who tested positive but did not have the specific cancer being screened for. Additionally, SCED-10 was applied to all adults in the target age group who were not eligible for recommended screening. The second supplemental screening scenario, called MCED-10, introduced a hypothetical blood test known as multi-cancer early detection (MCED) for the same ten cancers as SCED-10. The performance characteristics of an existing MCED test were used specifically for this subset of cancers. The third supplemental screening scenario represented an MCED test that is still under development. The performance characteristics used were based on a commercially available MCED test described in a previous study, which reported an aggregate TPR of 52% and an aggregate FPR of 0.4% in a US Surveillance, Epidemiology, and End Results (SEER) population of adults aged 50-79 years.[^15^](https://www.zotero.org/google-docs/?sLRDlR)

Our quantitative modeling and analysis show that TPR cannot be the most important metric for comparing screening tests. Instead, there must be multiple metrics that reflect different measures of population-level efficacy when designing a system that integrates a new approach to cancer detection with existing screening, as well as the administrative, economic, and ethical perspectives. Furthermore, our analysis supports a broader shift toward considering cancer as a molecular pathophysiology in the context of the individual person, rather than with a more narrow organ-by-organ approach. A future set of separate SCED tests might only be better suited to screen specialized cohorts of individuals at very high risk of certain cancer types.

Our modeling inputs used different definitions of test TPR for different cancer types, depending on available (often limited) data, typically based on retrospective case-control studies. For the purposes of simplicity, we assumed that detection was proportional to incidence, though this is not necessarily representative of test performance, and thus limits the generalizability of this study to real-world performance. Finally, our analysis estimates “pure performance” without considering natural history effects, which limits the ability of our model to predict system-level outcomes across each approach to screening.

*Calculating individuals who receive TPs and FPs*

Individuals receive separate tests for SCED systems, and the positivity rate was assumed independent for each separate test. The number of individuals receiving no positives (FPs) can then be calculated because they must jointly receive no positives from any set of tests they undergo. (This was computed separately for males and females due to sex-specific tests). The remaining individuals were then those that receive at least one positive (or false positive) from some test. Code/data is available at github link: <https://github.com/grailbio-publications/Madhavan_Screening_Systems>.

*Calculating positive predictive value (PPV)*

We calculated the PPV of each supplemental screening system according to test results, rather than individuals (i.e., one individual could receive more than one FP result within a single year).

We calculated the PPV of each system as follows:

**Total number of TP results / Total number of positive results**.

We calculated the PPVs using the same formula for each individual single-cancer test within the SCED-10 system, as summarized in **Supplemental Table 8**.

*Calculating aggregate negative predictive value (NPV)*

We calculated this metric by generalizing a single cancer NPV to reflect the proportion of subjects truly diagnosed as negative for the subset of eligible cancers tested, among all those who had negative test results for those cancers. We assume here that cancer risk was uncorrelated between cancer types for simplicity. The NPV is naturally a ratio, so aggregating multiple tests occurs by multiplying. Note that this calculation applies to individuals who receive negative results for all individual tests. As the set of cancers eligible for testing varies, the aggregate NPV is not directly comparable across systems because the rate of eligible cancers varies across screening systems.

**NPV_1_ = NPV_Cancer Subtype 1_ = TP_Cancer Subtype 1_ / (TN_Cancer Subtype 1_ + FN_Cancer Subtype 1_)**

**NPV_Aggregate_ = NPV_1_ × NPV_2_ × … × NPV_10_**

*Calculating all-cancer NPV*

We calculated this metric to reflect the system-level proportion of subjects who do not have any cancers (true negative for overall cancer), among all those who received a negative test result. For compatibility with aggregate NPV, we compute this similarly as a product, but taken over all cancer types. (Because cancer incidence was small, this was to first order the same as the sum.) We note that all-cancer NPV was distinct from aggregate NPV, because individuals are susceptible to developing untested cancers, and not testing does not mean safety. Because all-cancer NPV was not affected by the number of cancers tested, it can be compared across screening systems. Again, this measure applies to individuals receiving a negative test result, whether this was pooled (as in MCED systems) or a collection of individual tests (as in SCED systems).

**NPV_1_ = NPV_Cancer Subtype 1_ = TP_Cancer Subtype 1_ / (TN_Cancer Subtype 1_ + FN_Cancer Subtype 1_)**

**NPV_all_ = NPV_1_ × NPV_2_ × … × NPV_n_**

*Calculating number needed to screen (NNS)*

We defined the NNS as the number of tests performed (rather than individuals screened) to yield a true positive result.

We calculated the NNS of each system as follows:

**Total number of TP results / Total number of tests performed**.

# Supplemental Results

*Sensitivity analysis using the Galleri^®^ test performance characteristics*

When simulating MCED-10, we limited both systems to the same 10 specific cancer types to provide a fair comparison. However, MCED technology is designed to detect cancer signatures shared across multiple cancer types.^17^ In order to reflect MCED technology as intended, we also calculated the incremental impact of a third scenario using the performance of a commercially available MCED test for more than 50 types of cancer, Galleri^Ⓡ^ (labeled “MCED-G”) (aggregate TPR of 42% and FPR 0.49% in a SEER population of adults aged 50-79 years). Using the same approach as for SCED-10 and MCED-10 described both in the main manuscript and as above, the PPV and NNS for MCED-G were 46.29% and 234, respectively, both better than those for SCED-10 or MCED-10. Estimated costs of MCED-G were $99M with an assumed price of $949.^17^ Under a sensitivity analysis where the test cost of MCED-G was reduced by 25%, the incremental total cost for MCED-G was $74M. The commercially available MCED-G test would detect even more incremental cancers than the MCED-10 system, while maintaining the same low FPR. Our modeling showed that when added to routine care, in comparison to SCED-10 and MCED-10, the ideal approach to cancer screening is an MCED test that targets all cancer types (ie, MCED-G), because it detects more absolute cancers at a lower cost using a single test. These findings are summarized in comparison to the SCED-10, MCED-10, and USPSTF systems in Supplemental Figure 1 and Supplemental Tables 5-7.

# Supplemental Discussion

Our quantitative modeling and analysis show that TPR cannot be the most important metric for comparing screening tests. Instead, there must be multiple metrics that reflect different measures of population-level efficacy when designing a system that integrates a new approach to cancer detection with existing screening, as well as the administrative, economic, and ethical perspectives. Furthermore, our analysis supports a broader shift toward considering cancer as a molecular pathophysiology in the context of the individual person, rather than with a more narrow organ-by-organ approach. A future set of separate SCED tests might only be better suited to screen specialized cohorts of individuals at very high risk of certain cancer types.

Our modeling inputs used different definitions of test TPR for different cancer types, depending on available (often limited) data, typically based on retrospective case-control studies. For the purposes of simplicity, we assumed that detection was proportional to incidence, though this is not necessarily representative of test performance, and thus limits the generalizability of this study to real-world performance. Finally, our analysis estimates “pure performance” without considering natural history effects, which limits the ability of our model to predict system-level outcomes across each approach to screening.

**References**

[1. Hackshaw A, Cohen SS, Reichert H, Kansal AR, Chung KC, Ofman JJ. Estimating the population health impact of a multi-cancer early detection genomic blood test to complement existing screening in the US and UK. *Br J Cancer*. 2021;125(10):1432-1442. doi:10.1038/s41416-021-01498-4](https://www.zotero.org/google-docs/?hJau2C)

[2. A and B Recommendations | United States Preventive Services Taskforce. Accessed February 1, 2023. https://www.uspreventiveservicestaskforce.org/uspstf/recommendation-topics/uspstf-a-and-b-recommendations](https://www.zotero.org/google-docs/?hJau2C)

[3. Imperiale TF, Ransohoff DF, Itzkowitz SH, et al. Multitarget Stool DNA Testing for Colorectal-Cancer Screening. *N Engl J Med*. 2014;370(14):1287-1297. doi:10.1056/NEJMoa1311194](https://www.zotero.org/google-docs/?hJau2C)

[4. Kim JJ, Burger EA, Regan C, Sy S. Screening for Cervical Cancer in Primary Care: A Decision Analysis for the US Preventive Services Task Force. *JAMA*. 2018;320(7):706. doi:10.1001/jama.2017.19872](https://www.zotero.org/google-docs/?hJau2C)

[5. Pinsky PF, Gierada DS, Black W, et al. Performance of Lung-RADS in the National Lung Screening Trial: a retrospective assessment. *Ann Intern Med*. 2015;162(7):485-491. doi:10.7326/M14-2086](https://www.zotero.org/google-docs/?hJau2C)

[6. Lehman CD. National Performance Benchmarks for Modern Screening Digital Mammography: Update from the Breast Cancer Surveillance Consortium. *Radiology*. Published online April 2017. doi:10.1148/radiol.2016161174](https://www.zotero.org/google-docs/?hJau2C)

[7. Pasquinelli MM, Tammemägi MC, Kovitz KL, et al. Brief Report: Risk Prediction Model Versus United States Preventive Services Task Force 2020 Draft Lung Cancer Screening Eligibility Criteria—Reducing Race Disparities. *JTO Clin Res Rep*. 2021;2(3). doi:10.1016/j.jtocrr.2020.100137](https://www.zotero.org/google-docs/?hJau2C)

[8. Fedewa SA, Kazerooni EA, Studts JL, et al. State Variation in Low-Dose Computed Tomography Scanning for Lung Cancer Screening in the United States. *JNCI J Natl Cancer Inst*. 2021;113(8):1044-1052. doi:10.1093/jnci/djaa170](https://www.zotero.org/google-docs/?hJau2C)

[9. CDC - 2016 BRFSS Survey Data and Documentation. February 20, 2019. Accessed March 14, 2023. https://www.cdc.gov/brfss/annual_data/annual_2016.html](https://www.zotero.org/google-docs/?hJau2C)

[10. Landy R, Young CD, Skarzynski M, et al. Using Prediction-Models to Reduce Persistent Racial/Ethnic Disparities in Draft 2020 USPSTF Lung-Cancer Screening Guidelines. *J Natl Cancer Inst*. Published online January 5, 2021:djaa211. Epub ahead of print. doi:10.1093/jnci/djaa211](https://www.zotero.org/google-docs/?hJau2C)

[11. Berkowitz Z, Qin J, Smith JL, Saraiya M. Lack of Awareness of Human Papillomavirus Testing Among U.S. Women. *Am J Prev Med*. 2023;65(4):710-715. doi:10.1016/j.amepre.2023.03.024](https://www.zotero.org/google-docs/?hJau2C)

[12. Klein EA, Richards D, Cohn A, et al. Clinical validation of a targeted methylation-based multi-cancer early detection test using an independent validation set. *Ann Oncol*. 2021;32(9):1167-1177. doi:10.1016/j.annonc.2021.05.806](https://www.zotero.org/google-docs/?hJau2C)

[13. *Surveillance Epidemiology and End Results (SEER) Program. SEER*Stat Databases: November 2018 Submission. Incidence - SEER Research Data, 18 Registries, Nov 2019 Sub (2000-2017). Linked To County Attributes - Time Dependent (1990-2017) Income/Rurality, 1969-2017 Counties. National Cancer Institute; 2020.* Accessed April 15, 2020. www.seer.cancer.gov](https://www.zotero.org/google-docs/?hJau2C)

[14. 2023 USPSTF Recommendation Topics; Recommendation: Breast Cancer: Screening. Published online April 24, 2023. Accessed April 26, 2024. https://www.uspreventiveservicestaskforce.org/uspstf/draft-update-summary/breast-cancer-screening-adults](https://www.zotero.org/google-docs/?hJau2C)

[15. Liu MC, Oxnard GR, Klein EA, et al. Sensitive and specific multi-cancer detection and localization using methylation signatures in cell-free DNA. *Ann Oncol*. 2020;31(6):745-759. doi:10.1016/j.annonc.2020.02.011](https://www.zotero.org/google-docs/?hJau2C)

[16. Hubbell E, Clarke CA, Aravanis AM, Berg CD. Modeled Reductions in Late-stage Cancer with a Multi-Cancer Early Detection Test. *Cancer Epidemiol Biomark Amp Prev*. 2021;30(3):460. doi:10.1158/1055-9965.EPI-20-1134](https://www.zotero.org/google-docs/?hJau2C)
